# Supplementary material for: Design and fabrication of 3D-printed patient-specific soft tissue and bone phantoms for CT imaging
Source: Sci Rep. 2023 Oct 15;13:17495. doi: 10.1038/s41598-023-44602-9 (PMC10577126; doi:10.1038/s41598-023-44602-9)
Supplement: Supplementary file 1 — Supplementary Figures. [file 41598_2023_44602_MOESM1_ESM.docx]

**Supplemental Figures**

|  | a |  |
| --- | --- | --- |


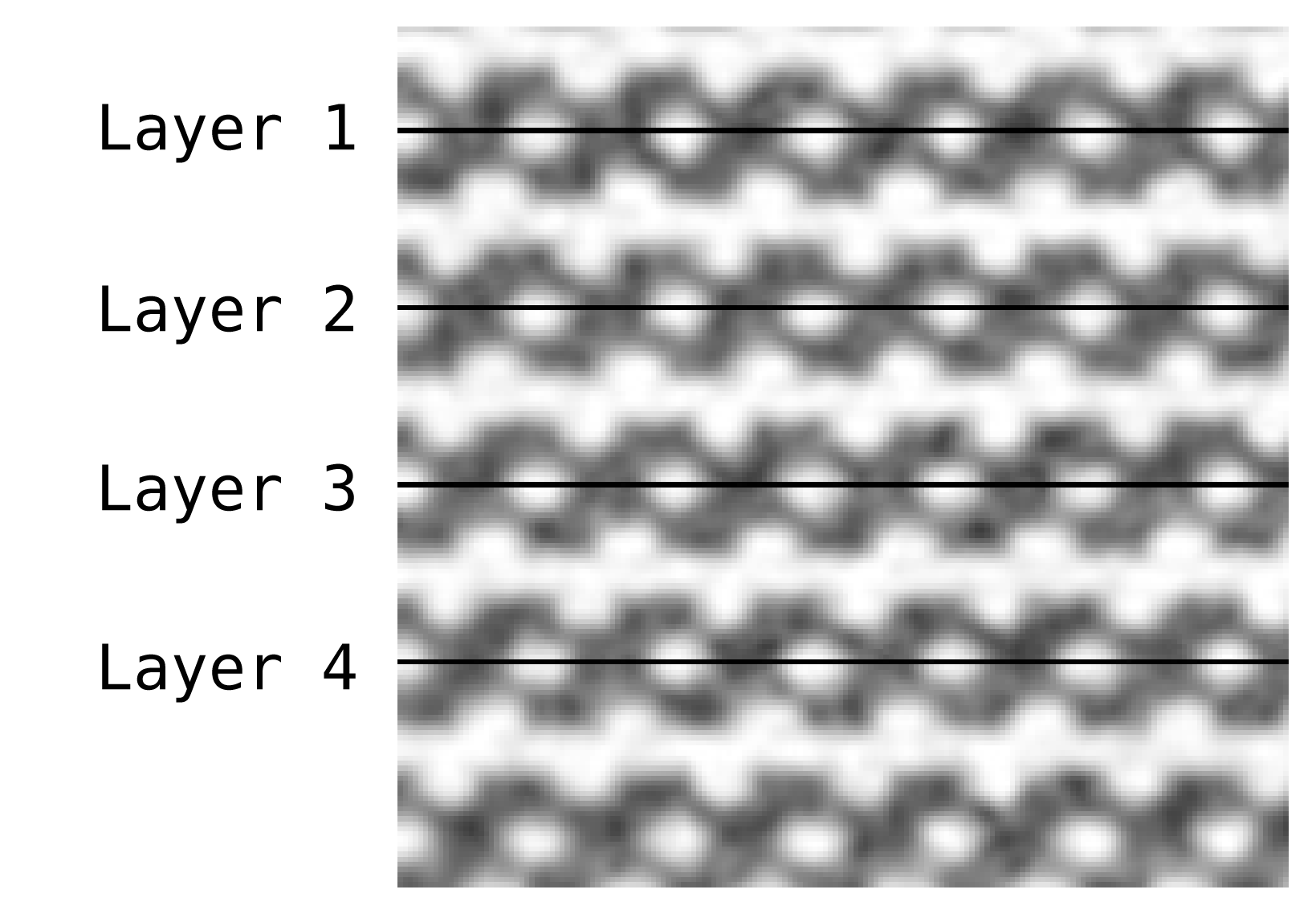


| b | c |
| --- | --- |


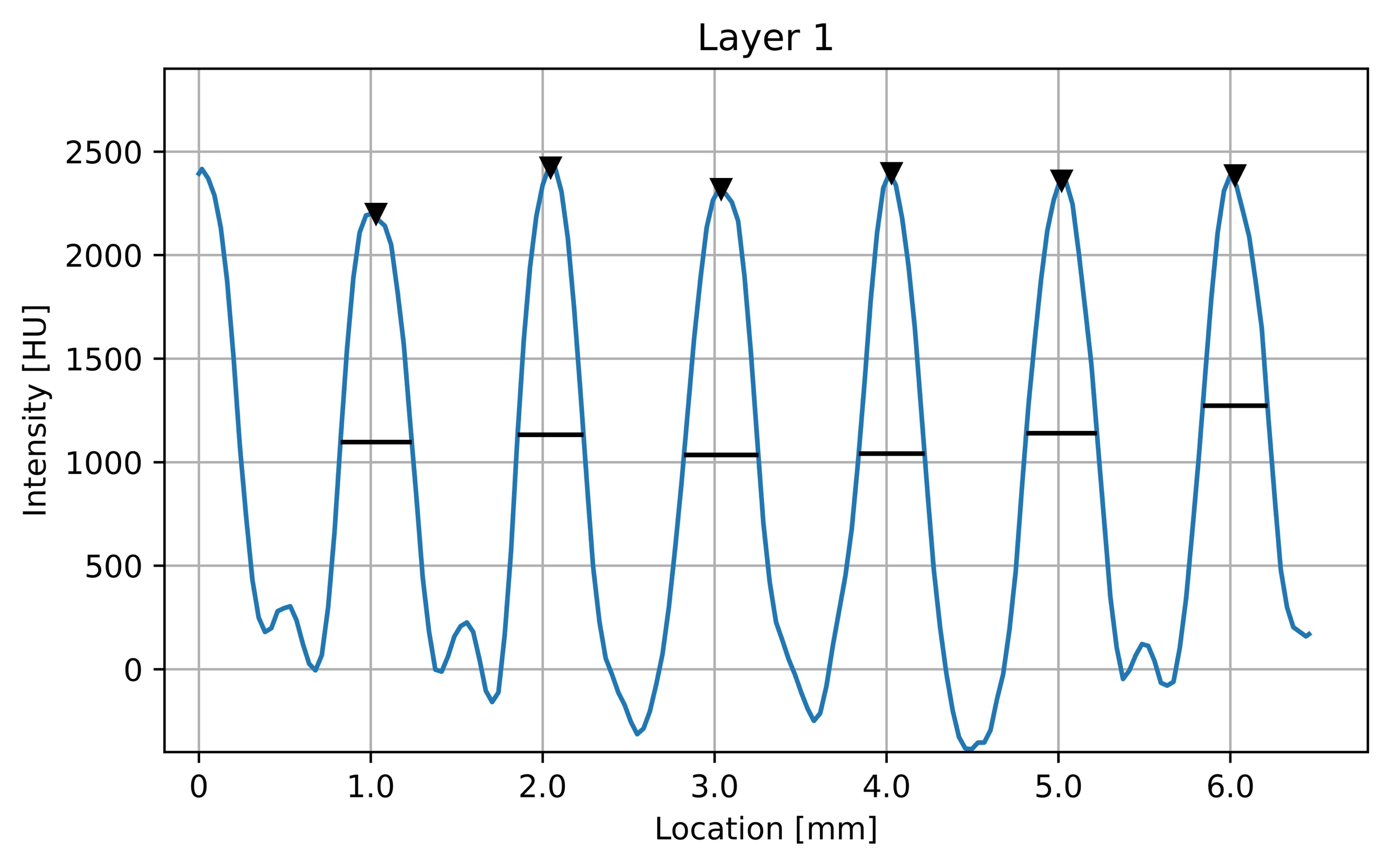

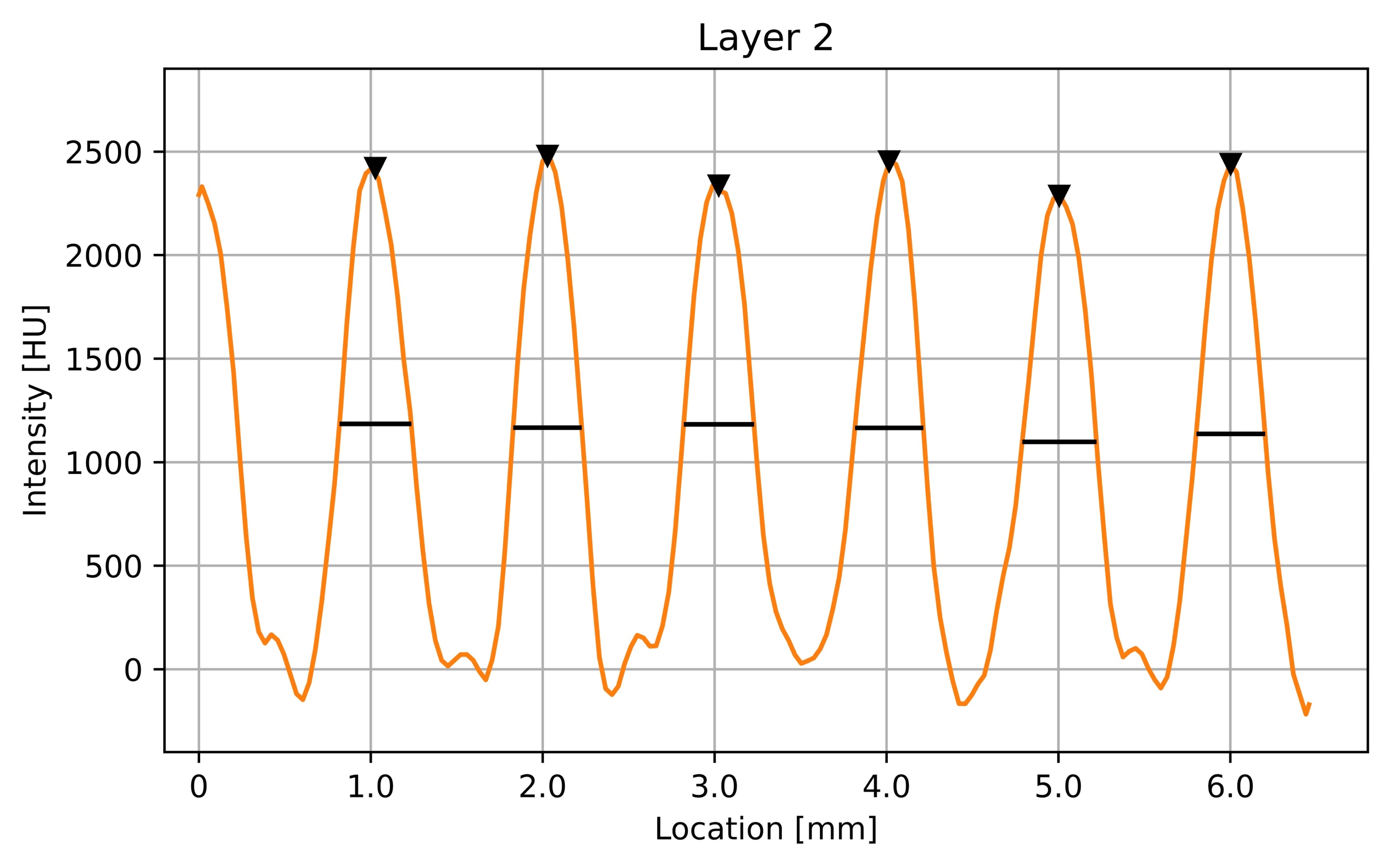


| d | e |
| --- | --- |


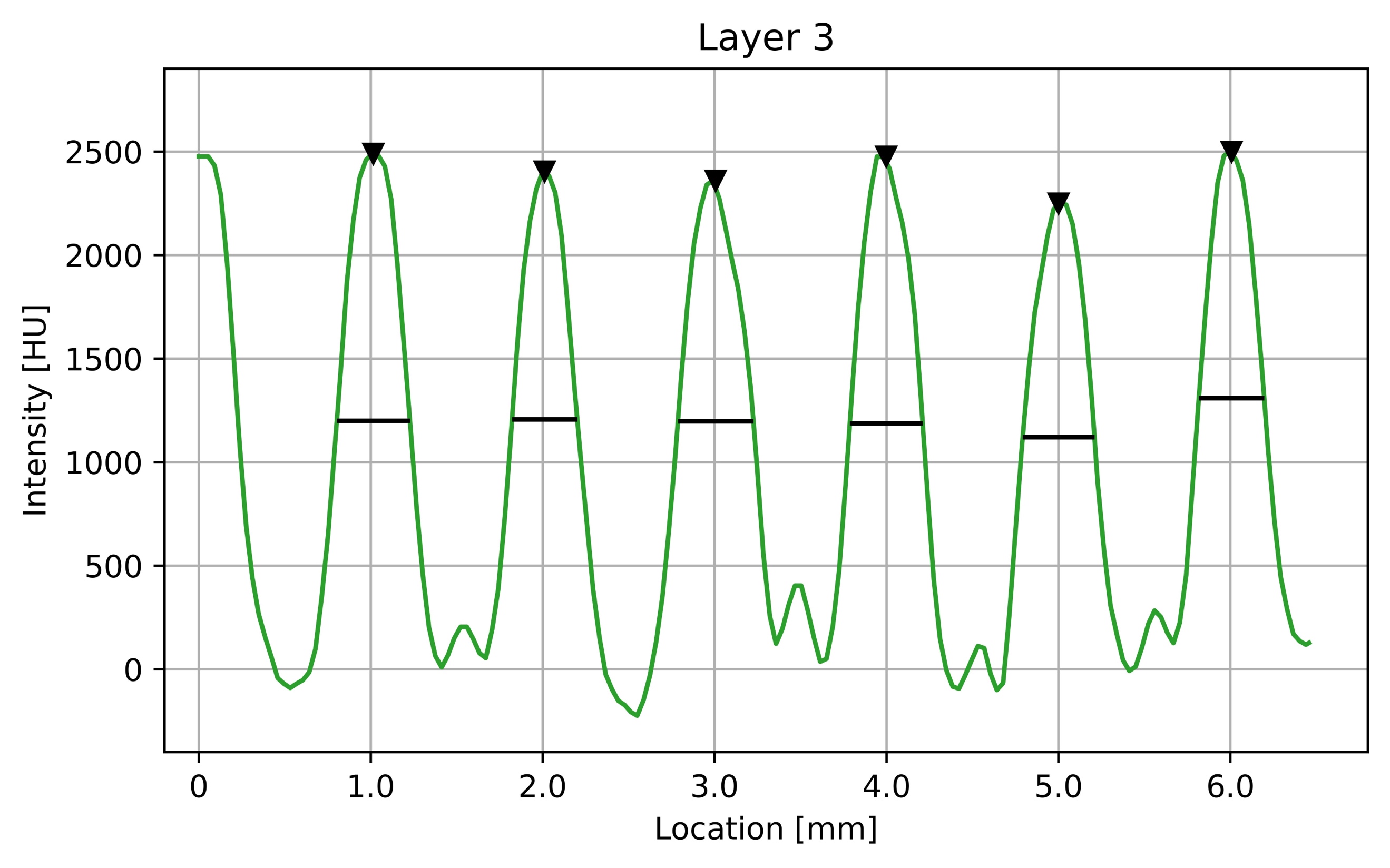

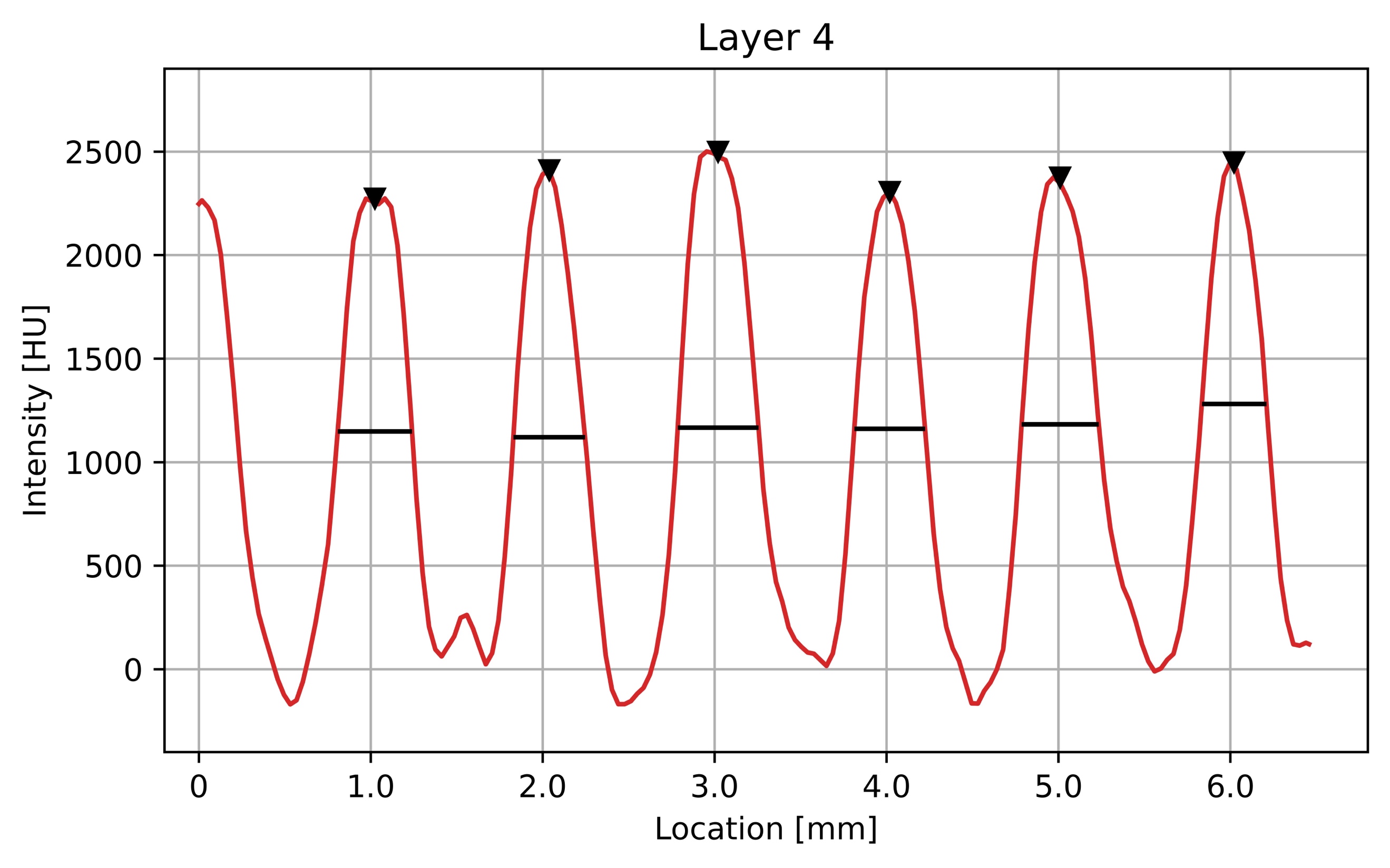


**Supplemental Figure 1. Comparison of filament lines in different layers.** In Figure (a), an orthogonal micro-CT view of a phantom with a 50% infill. The black lines indicate the positions of selected line profiles, as shown in Figures (b) to (e). These positions were manually chosen and approximated to represent the center of each layer.

**Supplemental Table 1. Measured filament line spacing and line width in four layers.**

|  | Filament line spacing | | Filament line width | |
| --- | --- | --- | --- | --- |
|  | mean | ± stddev | mean | ± stddev |
| Layer 1 | 0.9994 | ± 0.011 | 0.4001 | ± 0.021 |
| Layer 2 | 0.9950 | ± 0.004 | 0.4092 | ± 0.012 |
| Layer 3 | 0.9983 | ± 0.005 | 0.4109 | ± 0.023 |
| Layer 4 | 0.9997 | ± 0.012 | 0.4259 | ± 0.030 |
| *Overall* | *0.9981* | *± 0.008* | *0.4115* | *± 0.022* |

The centers of the filament lines are determined at the midpoint of the width at half maximum of the peaks in the line profile of each layer, as indicated by triangles. Subsequently, the filament line spacings (N = 5) are estimated as the distance between adjacent triangle markers. The widths of the filament lines (N = 6) are measured as the half-maximum width. Prior to measurement, the images were manually resliced and resampled to ensure correct positioning. Line profiles were resampled to 1 data point per 1 μm (10^-6^ m) before the measurement.

| a | b | c |
| --- | --- | --- |


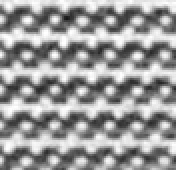

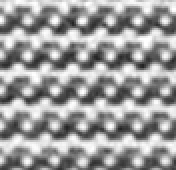

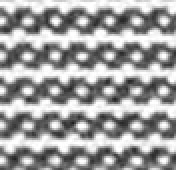


| d | e | f |
| --- | --- | --- |


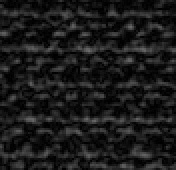

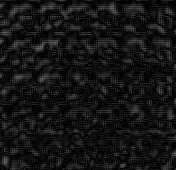

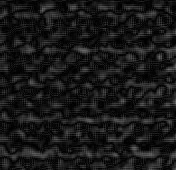


**Supplemental Figure 2. Comparison of three micro-CT phantoms.** (a)-(c) are manually allied regions of the same section in three separately printed phantoms. Window level/width are -750/3500 HU. (d)-(f) are absolute values of difference images between these three images, (a) vs. (b), (a) vs. (c), and (b) vs. (c), respectively. Window level/width are 1750/3500 HU.
